# Exploring the mechanism of Kanglixin capsules in the treatment of non-small cell lung cancer based on network pharmacology and molecular docking

**DOI:** 10.1097/MD.0000000000044768

**Published:** 2025-09-26

**Authors:** Yilong Li, Mingxuan Li, Xu Gong, Kangwei Xia, Zhaorui Liu, Xiangjun Qiu

**Affiliations:** a College of Basic Medicine and Forensic Medicine, Henan University of Science and Technology, Luoyang City, Henan Province, China.

**Keywords:** Kanglixin capsule, molecular docking, network pharmacology, non-small cell lung cancer

## Abstract

This study explores the mechanism of action of KangLiXin capsule (KLX) in the intervention of non-small cell lung cancer (NSCLC) using network pharmacology and molecular docking methods to identify its potential targets and pathways, provide new directions for experimental research on the prevention and treatment of NSCLC with KLX, and offer scientific evidence for its clinical application. The chemical composition was collected through Traditional Chinese Medicine Systems Pharmacology, Herb and domestic and international authoritative databases, supplemented with the data and validated the structure of Simplified Molecular Input Line Entry System with Pubchem, and predicted the targets in the Swisstargetprediction platform. Meanwhile, NSCLC-related target information was collected and standardized in GeneCards, Online Mendelian Inheritance in Man and Drugbank disease databases, and Wayne diagrams were drawn to show the overlap and discrepancy in the Microbiology Letter platform. Next, the intersecting targets of KLX and NSCLC were constructed into a protein–protein interaction network to identify the core targets and visualized in Cytoscape 3.10.0. Based on the identified targets, a “drug-active ingredient-target” network model was constructed. The intersected targets were imported into Database for Annotation, Visualization and Integrated Discovery for gene ontology annotation and Kyoto encyclopedia of genes and genomes pathway analysis and visualized in WBC. Finally, molecular docking was carried out between the screened core target and the corresponding active ingredient, and the binding energies were calculated and the two-dimensional and three-dimensional model diagrams of molecular docking were drawn. A total of 66 active compounds were screened from KLX, and 688 related gene targets were identified. We obtained 1379 disease targets of NSCLC and 187 intersecting targets. The gene ontology analysis yielded 1145 entries covering biological processes, cellular components, and molecular functions. The Kyoto encyclopedia of genes and genomes analysis revealed 178 pathways, with the most critical one being the NSCLC pathway (hsa05223). In addition, molecular docking showed that the representative compounds in KLX were able to bind stably to some of the core target proteins of NSCLC, exhibiting strong binding activity. Network pharmacological analysis revealed the complex regulatory relationship of KLX. Molecular docking experiments verified the stable binding of the active compounds in KLX to the targets.

## 1. Introduction

Lung cancer is the leading cause of morbidity and mortality of malignant tumors worldwide, but the specific molecular mechanisms of its development have not been fully understood. The development of non-small cell lung cancer (NSCLC) is often accompanied by complex changes, such as metastasis, gene mutation, and multidrug resistance. Despite recent advances in our understanding of the risk factors, disease progression, immunomodulation, and therapeutic options for NSCLC, NSCLC remains the leading cause of cancer death.^[1]^ A number of important advances in the treatment of NSCLC in recent years, such as targeted and immunotherapies, but the overall survival of patients has not reached the desired level and is accompanied by problems such as drug resistance and side effects. This is mainly due to the fact that the initial symptoms of NSCLC are often hidden and not easily detected, and invasive and distant metastases may occur at an early stage, resulting in many patients being in the middle and late stages of the disease by the time they are detected. The high recurrence rate in the middle and late stages of NSCLC poses a great challenge to the life safety of patients. Therefore, we should always adhere to the principles of early detection, early diagnosis and early treatment in the prevention and treatment of NSCLC in order to improve the survival rate and quality of life of patients.^[2]^ Exploring new therapeutic strategies and drug targets is essential to improve the therapeutic effects of NSCLC.

Kanglixin capsule is a major innovation in the field of medicine in recent years, and has attracted widespread attention in the field of tumor therapy for its unique antimetabolic mechanism. As a novel drug, the core effect of KangLiXin (KLX) capsules is to inhibit the activity of thymidylate synthase, and the blockage of this key step directly affects the metabolic process of tumor cells, thus significantly slowing down the development of tumors^[3]^ and bringing substantial therapeutic effects to tumor patients.

The herbal ingredients in Kanglixin capsules are not simply combined haphazardly but carefully formulated to maximize their therapeutic efficacy. Ingredients such as Ferulae Resina (ferula), Coridius Chinensis (stink bug), and Caryophylli Flos (clove) in the medication have the function of tonifying the body and expelling pathogens, thereby enhancing the body’s immune system and resisting the invasion of tumor cells. Ingredients such as Rhei Radix et Rhizoma (rhubarb), Curcumae Longae Rhizoma (turmeric) have the effect of softening hardened masses and dispersing nodules, directly acting on tumor tissue to promote its softening and dissolution. Herbs like Chebulae Fructus (medicine terminalia), Ophiocordyceps sinensis (cordyceps) help regulate the body’s metabolic functions, enhancing the efficacy of the medication.

**Figure 1.**
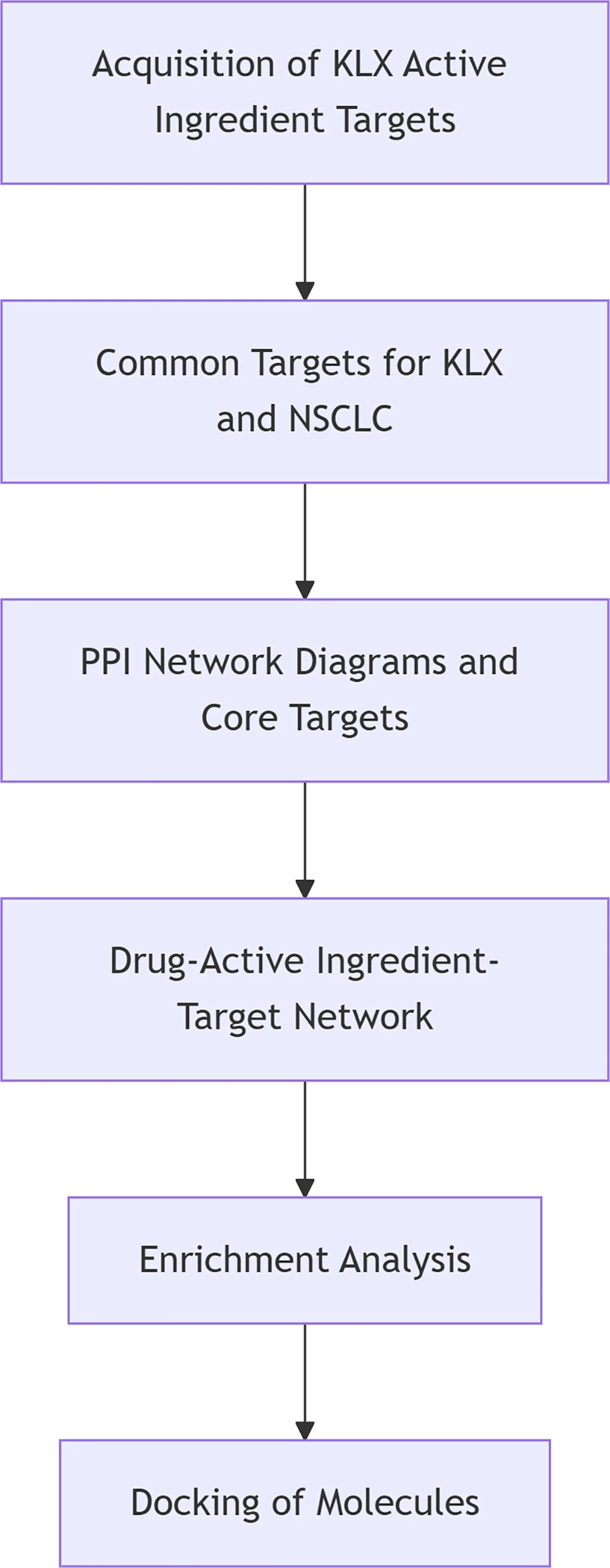
Flowchart of this study.

This study aims to utilize network pharmacology and molecular docking technology to explore the potential mechanism of action of Kanglixin capsules in the treatment of NSCLC. First, we will collect information on the chemical components of Kanglixin capsules and utilize the database resources to predict the possible targets of these components. Subsequently, we will analyze the relationship between these targets and NSCLC related signaling pathways, and construct a drug-target-pathway network diagram to reveal the possible mechanism of action of Kanglixin capsules in the treatment of NSCLC. Meanwhile, the interactions between the active ingredients and the predicted targets will be further verified by molecular docking technology to ensure the reliability of the mechanism of action. The network pharmacology and molecular docking technology adopted in this study will also provide new methods and ideas for the study of the mechanism of action of other Chinese medicinal preparations. This will help promote the development and application of Chinese medicinal preparations in the modern medical system and bring benefits to more patients. The flowchart of this study is summarized in Figure 1.

## 2. Materials and methods

### 2.1. Collection of KLX-active compounds and prediction of related targets

The Traditional Chinese Medicine Systems Pharmacology database was used to systematically collect the chemical compositions of rhubarb, turmeric, Aucklandiae Radix (aucklandia lappa), medicine terminalia, cordyceps, and clove in KLX, and the corresponding target information was organized. Then, the Herb database was utilized and combined with authoritative databases at home and abroad, such as China Knowledge, Wanfang, Pubmed, and Web of Science, to obtain the chemical compositions of ferula and stink bug. The collected chemical compositions (ferula and stink bug) were verified by the Pubchem database and organized to obtain their corresponding Simplified Molecular Input Line Entry System structures. Finally, the structures were entered into the Swisstargetprediction platform to obtain the target information of the corresponding compounds.

The criteria for active compound screening were established as follows. Oral bioavailability ≥ 30% and drug-likeness ≥ 0.18. At least 2 “Yes” outcomes from drug-likeness prediction rules (Lipinski, Ghose, Veber, Egan, Muegge). Probability > 0 in results from the SwissTargetPrediction database.

### 2.2. NSCLC-related target mining

The keywords “Non-small Cell Lung Cancer” and “NSCLC” were used to access and collect disease-related targets in the following 3 public databases: GeneCards, Online Mendelian Inheritance in Man, and Drugbank to obtain and collect disease-related targets. The obtained target proteins were converted by Uniprot database, and the obtained targets were plotted as Wayne diagrams in the microbiology platform.

### 2.3. Protein–protein interaction (PPI) network construction and core target screening

To further identify the coregulation targets, we submitted the KLX-NSCLC intersection targets to the STRING database. When setting the parameters, “Homo sapiens” was selected as the species, and the lowest interaction score was set to medium confidence (0.400), and the rest of the parameters were kept as defaults to obtain the PPI map. Subsequently, we imported the PPI results into Cytoscape3.10.0 software (Free Software Foundation , Inc, Boston) and selected analyze network, and then set the target size, font size, transparency, and color shades based on the degree value to visualize the network.

### 2.4. Construction of drug-active ingredient-target network

In order to elucidate the potential mechanism of action of KLX against NSCLC, we created a network model and its related tape files based on the identified targets. Subsequently, a drug-active ingredient-target interaction network was successfully constructed using Cytoscape 3.10.0 software. In this network, the nodes visually represent the active ingredients or targets, and the associations between them are demonstrated by connecting lines.

### 2.5. Enrichment analysis

The data were imported into the database for annotation, visualization and integrated discovery database for gene ontology (GO) function annotation and Kyoto encyclopedia of genes and genomes (KEGG) pathway enrichment analysis. In the GO analysis, we focused on the top 10 entries of biological process (BP), molecular function (MF), and cell component. Genes for further study were selected based on a consequence threshold of *P* < .05, including the top 10 from the GO analysis and the top 20 from the KEGG pathway analysis. In order to display these results more intuitively, we visualized them on the Microbiology platform to reveal the BPs and metabolic pathways involved in the intersected targets.

### 2.6. Docking of molecules

In order to verify the accuracy of the results of the preliminary network pharmacological screening and to provide strong support for the subsequent pharmacological studies and drug design, the top 10 core targets and their corresponding compounds were selected for molecular docking simulations. In the field of pharmacology, the threshold of binding energy is usually considered to be crucial for judging the strength of ligand–receptor interactions.^[4]^ Specifically, a binding energy of <−5.0 kcal/mol indicates good binding, whereas a binding energy of <−7.0 kcal/mol is considered strong binding activity. These thresholds are useful for assessing the strength of ligand–receptor interactions. In this study, a binding energy of <−7.0 kcal/mol was set as a strict criterion for screening.

For docking analysis, we downloaded the two-dimensional structure data of the potentially active compounds in KLX from the PubChem database and obtained the three-dimensional structures of the target proteins from the PDB database, which were saved in PDB format. In AutoDock4 software (WordPress, San Francisco), we preprocessed the protein files, including removing water molecules and replacing them with hydrogen atoms, then designated them as receptors and converted them to protein receptor files in PDBQT format. In addition, we cleaned up the protein structure by removing water molecules and minor ligands using Pymol2 software (BioLuminate, Schrödinger, LLC, New York), and then imported these data into AutoDockTools 1.1.2 for further preprocessing for dehydration reactions and hydrogenation.

Finally, in order to analyze in detail the linkage and potential activity between the target and KLX molecules, molecular docking simulations were performed using AutoDock vina1.2 software. The aim of this step was to validate the accuracy of the preliminary network pharmacological screening results and to provide a strong support for subsequent pharmacological studies and drug design.

## 3. Results

### 3.1. Acquisition of KLX active ingredient targets

After rigorous screening and elimination of unnecessary target chemistry, we further supplemented the information on the core drug components of KLX obtained from the literature. After these processes, we finally identified a series of active ingredients and 66 active compounds of KLX. In total, there were 5 active compounds of clove, including kamphiol and quercetin; 4 active compounds of cordyceps, including arachidonic acid and linolenic acetate; 7 active compounds of chebulin, including crushed leaf cordycepin and pellagracin; 10 active compounds of rhubarb, including β-sitosterol and rhubarbol methyl ether glucoside; 3 active compounds of curcuma longa, including stigmasterol and canola stigmasterol; and 5 active compounds of wood flavors, including betulinic acid and stigmasterol. There are 19 active compounds of stink bug such as adenine and cordycepin, and 13 active compounds of ferula such as ferulic acid and broadleaf ferulone. The detailed composition is shown in Table 1.

**Table 1:** The active ingredients and compounds of KLX.

Composition	Number	Active compound	Molecular formula	Relative molecular mass (g/mol)
clove	DX01	Kaempferol	C15H10O6	286.24
	DX02	Quercitrin	C15H10O7	302.23
	DX03	ZINC03860434	C24H38O4	390.62
	B1	β-Sitosterol	C29H50O	414.7
	DX04	Strictosamide_qt		498.52
cordyceps	DCXC01	Arachidonic acid	C20H32O2	304.5
	DCXC02	Ethyl linolenate	C20H36O2	308.5
	B1	β-Sitosterol	C29H50O	414.7
	C1	Cholesterol	C27H46O	386.7
medicine terminalia	HZ01	Cheilanthifoline	C19H19NO4	325.4
	HZ02	(R)-(6-Methoxyquinolin-4-yl)-[(1R,2R,4R,5S)-5-vinylquinuclidin-2-yl] methanol	C20H24N2O2	324.4
	HZ03	Peraksine	C19H22N2O2	310.4
	HZ04	Ellipticine	C17H14N2	246.31
	HZ05	Chebulic acid	C14H12O11	356.24
	HZ06	7-Dehydrosigmasterol	C29H50O	414.79
	HZ07	Ellagic acid	C14H6O8	302. 19
rhubarb	DH01	Daucosterol qt		386.73
	B1	β-Sitosterol	C29H50O	414.7
	DH02	Physcion-1-O-β-D-glucoside	C28H32O15	608.5
	DH03	8-O-[β-D-glucopyranosyl-6-O-oxaloyl]cassialactone	C22H24O12	480.46
	DH04	Emodin-1-O-β-D-glucopyranoside	C21H20O10	432.4
	DH05	Toralactone	C15H12O5	272.25
	DH06	Rhein	C15H8O6	284.22
	DH07	Catechin	C15H14O6	290.27
	DH08	Eupatin	C18H16O8	360.3
	DH09	Aloe barbadensis	C15H10O5	270.24
	S1	Stigmasterol	C29H48O	412.7
turmeric	JX01	Campesterol	C28H48O	400.7
	C1	Cholesterol	C27H46O	386.7
aucklandia lappa	MX01	Betulinic acid	C30H48O3	456.7
	S1	Stigmasterol	C29H48O	412.7
	MX02	β-Sitosterol	C29H50O	414.7
	MX03	Oleandrin	C19H22O6	346.4
	MX04	7H-benzo [c]carbazole	C16H11N	217.26
nux vomica	JXC01	Adenine	C5H5N5	135.13
	JXC02	2′-O-methyluridine	C10H14N2O6	258.23
	JXC03	Adenosine	C10H13N5O4	267.24
	JXC04	3′-Deoxyadenosine	C10H13N5O3	251.24
	JXC05	L-phenylalanine	C9H11NO2	165.19
	JXC06	L-asparagine	C20H20N2O6	384.4
	JXC07	Oleic acid	C18H34O2	282.5
	JXC08	Arachidonic acid	C20H40O2	312.5
	JXC09	Choline	C5H14NO+	104.17
	JXC10	Nicotine	C10H14N2	162.23
	JXC11	Nicotinamide	C6H6N2O	122. 12
	JXC12	Indoline-3-carboxylic acid	C9H7NO2	161.16
	JXC13	Indole-3-carboxaldehyde	C9H7NO	145.16
	JXC14	Indoline-3-carboxylic acid	C9H7NO2	161.16
	JXC15	Kynruenic acid	C10H7NO3	189.17
	JXC16	3,4-Dihydroxypropiophenone	C9H10O3	166.17
	JXC17	3,4-Dihydroxyphenylacetic acid	C8H8O4	168.15
	JXC18	Vanillic acid	C8H8O4	168.15
	JXC19	Cyclo	C17H21N3O2	299.37
ferula	AW01	Ferulic acid	C10H10O4	194.18
	AW02	Conferone	C24H28O4	380.5
	AW03	Conferol	C24H30O4	382.5
	AW04	Assafoetidin	C24H30O4	382.5
	AW05	3-Carene	C10H16	136.23
	AW06	Alpha-pinene	C10H16	136.23
	AW07	Farnesol	C15H26O	222.37
	AW08	Assafoetidin	C24H30O4	382.5
	AW09	Kaempferol A	C24H30O4	382.5
	AW10	Kaempferol B	C24H30O4	382.5
	AW11	Kaempferol C	C24H30O4	382.5
	AW12	Ferulenol A	C26H32O7	456.5
	AW13	Confertifolin	C15H22O2	234.33

clove = Caryophylli Flos, cordyceps = Ophiocordyceps sinensis, ferula = Ferulae Resina, KLX = Kanglixin capsule, medicine terminalia = Chebulae Fructus, rhubarb = Rhei Radix et Rhizoma, turmeric = Curcumae Longae Rhizoma.

### 3.2. Common targets for KLX and NSCLC

Through an in-depth search of the UniProt database, we screened the gene–protein data under specific conditions for 6 active ingredients: rhubarb, turmeric, aucklandia lappa, medicine terminalia, cordyceps, and clove. After selecting human for popular organisms and reviewed (Swiss-Prot) for status, we removed duplicates and obtained the following results: 65 gene targets related to active ingredients of rhubarb, 32 for turmeric, 31 for aucklandia lappa, 70 for medicine terminalia, 76 for cordycep, and 183 for clove. Subsequently, we identified 273 ferula gene targets and 299 cordyceps gene targets in the SwissTargetPrediction database. Next, we summarized all the identified gene targets and removed duplicates, and finally obtained a total of 688 KLX gene targets.

**Figure 2.**
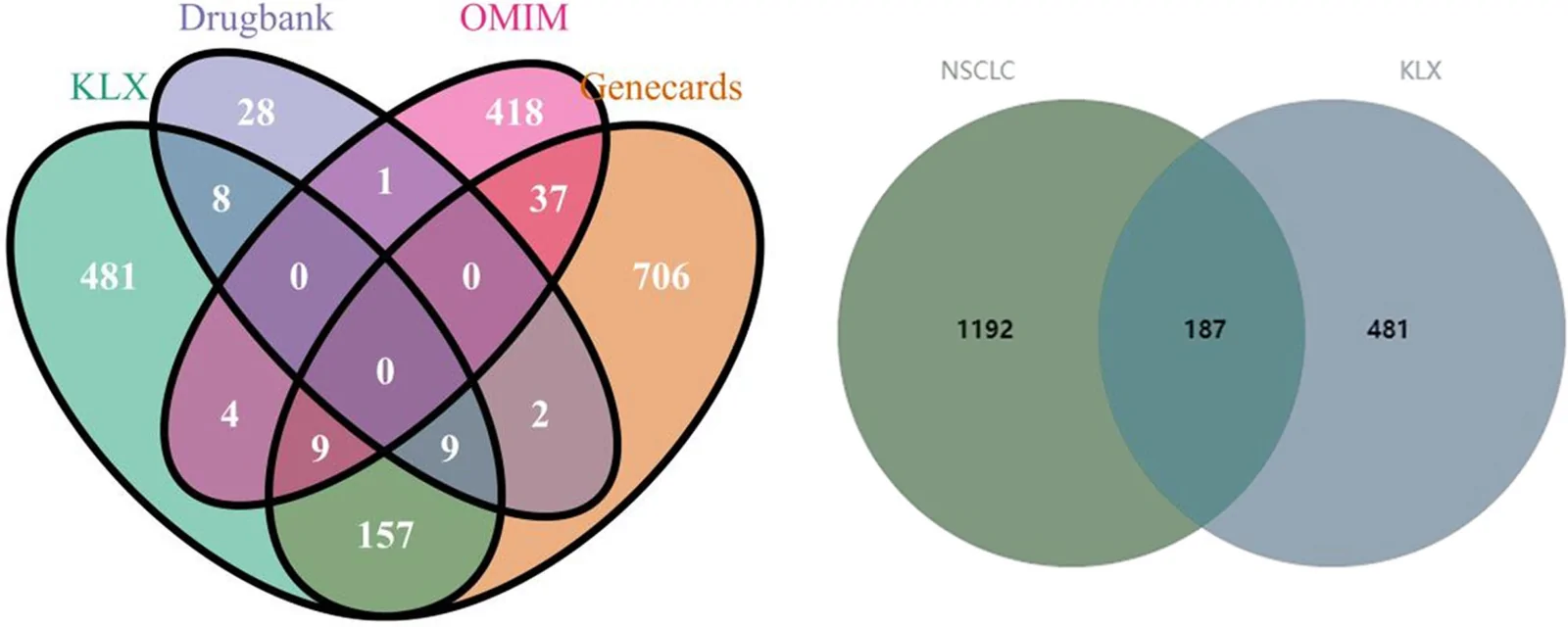
The cross-target map between KLX active targets and NSCLC targets (obtained from 3 disease databases) (L) and intersection target map of KLX with active ingredients and NSCLC(R). (L) The cross-target map between KLX active targets and NSCLC targets (obtained from 3 disease databases). The KLX-related genes by taking a union of all the results from databases. (R) Intersection target map of KLX with active ingredients and NSCLC. The KLX–NSCLC shared target genes by taking an intersection of AR target genes and NSCLC-related genes. KLX = Kanglixin capsule, NSCLC = non-small cell lung cancer.

During the search for NSCLC disease-related targets, we screened 7364 disease genes from the GeneCards database. In order to further refine the results, we screened the relevance score at the median and removed duplicates for 2 consecutive times, and finally obtained 920 disease genes. In addition, we also obtained disease gene data from the Online Mendelian Inheritance in Man database and the Drugbank database, and obtained 469 and 48 disease genes after de-duplication, respectively. Finally, we compared the disease targets (1379 in total) obtained from these 3 disease databases with the drug targets, and plotted the intersecting Wayne diagrams (Fig. 2) to show the commonalities between them.

### 3.3. PPI network diagrams and core targets

**Figure 3.**
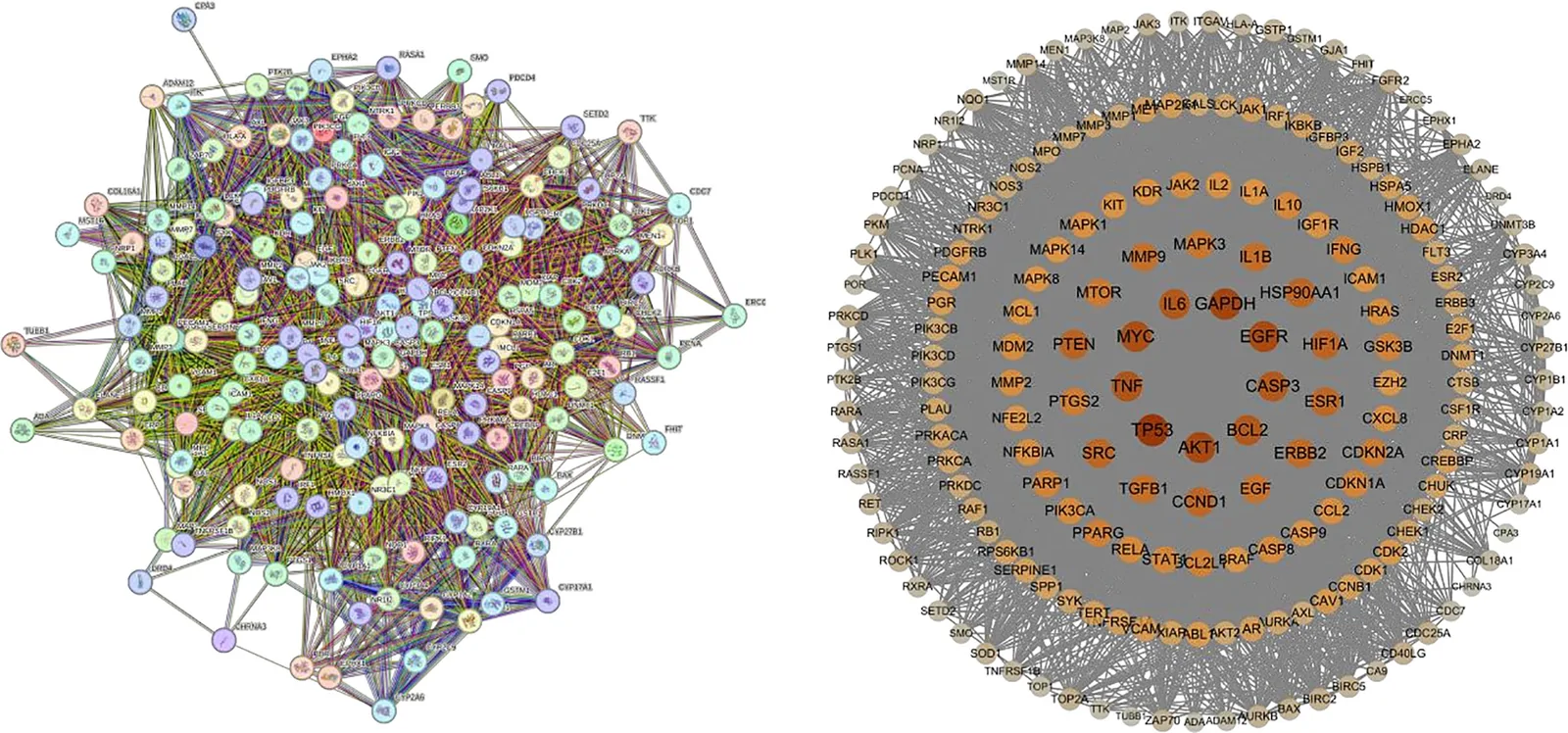
PPI image obtained from STRING (L) and PPI image visualized by Cytoscape (R). (L) PPI image obtained from STRING. (R) PPI image visualized by Cytoscape. In this network, nodes represent proteins, with colors transitioning from dark to light to indicate the strength of binding interactions among them. Edges illustrate the associations between different proteins. PPI = protein–protein interaction.

Using the STRING database, we successfully constructed a PPI network diagram containing 187 potential regulatory targets (Fig. 3). Subsequently, we imported the TSV file generated by STRING into Cytoscape 3.10.0 software and used its excellent visualization capabilities to further generate a detailed PPI network diagram (Fig. 3), which consisted of 187 nodes and 5833 edges. After carefully analyzing the PPI network diagram, we found that the degree values of several protein targets, such as EGFR and IL-6, were significantly higher than the average, which showed their close connection and strong interaction with other targets in the network.

In order to highlight the importance of these key targets in KLX treatment of NSCLC, we listed the top 10 core targets based on the degree value (Table 2). These core targets occupy a central position in the PPI network and are strongly associated with multiple active ingredients, further reinforcing their central role in KLX treatment of NSCLC.

**Table 2:** The top 10 core targets screened by Cytoscape 3.10.0.

Degree	Target protein	Corresponding active compounds
165.0	TP53	Aloe-emodin
156.0	GAPDH	Adenosine
151.0	EGFR	(R)-(6-Methoxy-4-quinolyl)-[(2R,4R,5S)-5-vinylquinuclidin-2-yl] methanol
152.0	AKT1	Kaempferol
		Quercetin
140.0	CASP3	Arachidonic acid
		Beta-sitosterol
147.0	MYC	Aloe-emodin
141.0	BCL2	Beta-sitosterol
142.0	TNF	Aspongopusamide B
		Aloe-emodin
		Kaempferol
		Quercetin
141.0	IL6	Quercetin
132.0	PTEN	Quercetin

### 3.4. Drug-active ingredient-target network

**Figure 4.**
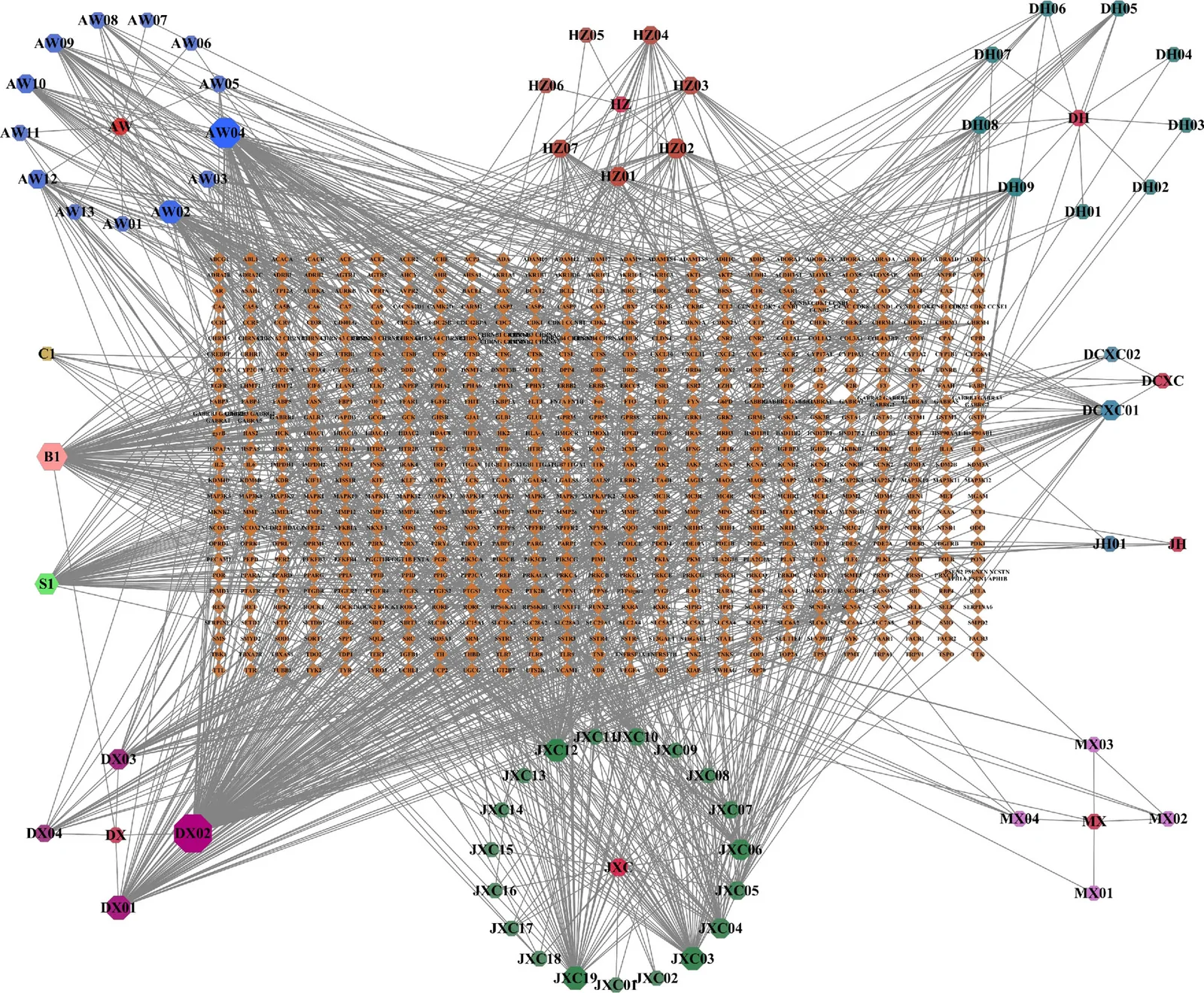
Visualized drug active ingredient target map. Illustration showing the network interactions between target genes and their associated signaling pathways.

The organized network and tape files were imported into Cytoscape 3.10.0 to draw the drug-active ingredient-target maps (Fig. 4). The red circles represent 8 drugs, and the corresponding active compounds are surrounded by the drugs, with the larger ones indicating the highest degree of association, for example, Aw04 weißin, DH02 rhododendron methyl ether glucoside, JXC03 adenosine, JXC12 indole-β-carboxylic acid, HZ01 crushed leaf corynoline, and DH09 aloe vera rhododendron. This diagram shows the relationship between the 3 in a very intuitive way.

### 3.5. Enrichment analysis

**Figure 5.**
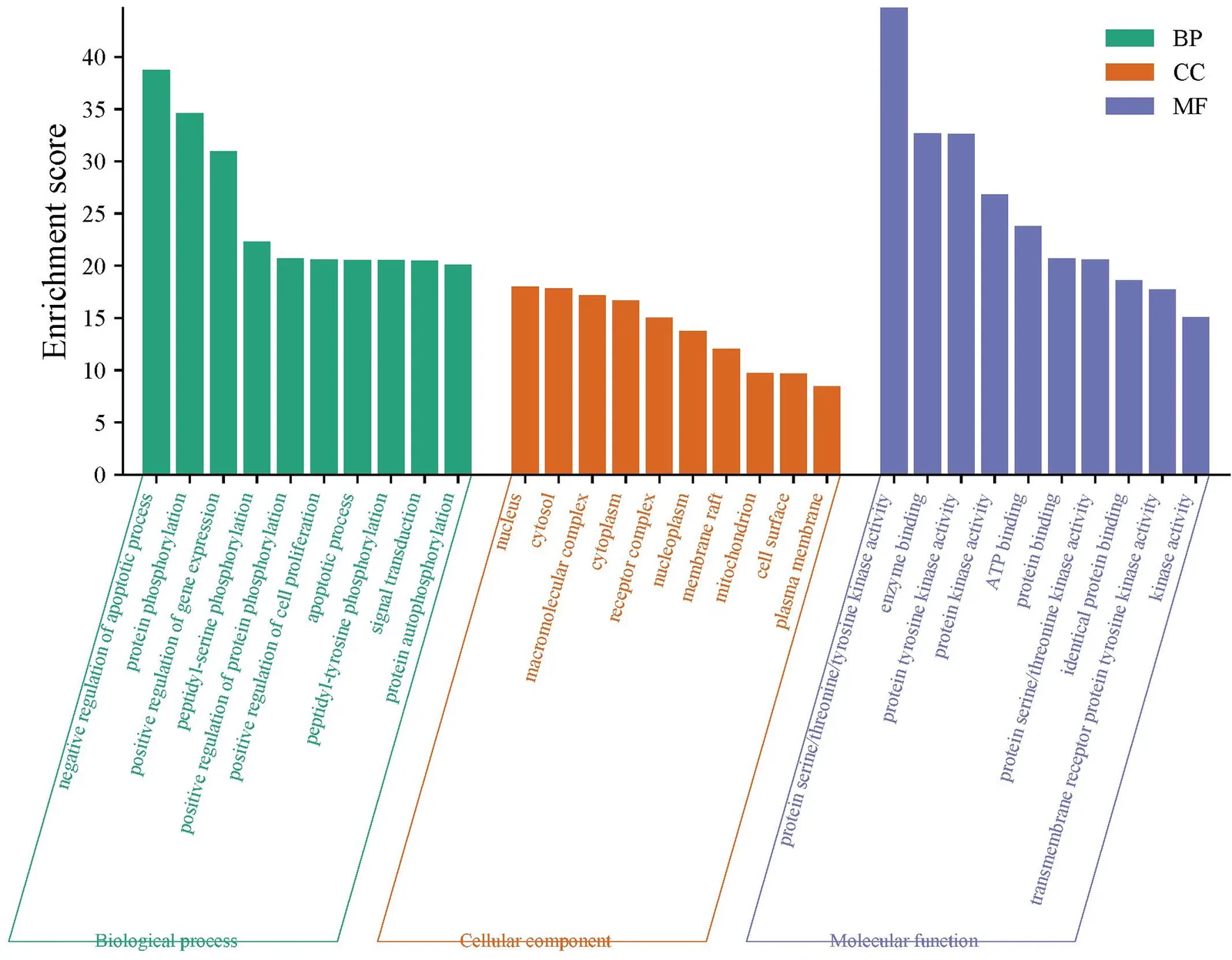
Visualized drug active ingredient target map.

As shown in Figure 5, a total of 1145 statistically significant GOs were obtained in this study, including 857 BPs, 112 cell components, and 176 MFs, and the targets of KLX for NSCLC were mainly enriched in BPs such as negative regulation of apoptosis (GO:0043066), protein phosphorylation (GO:0006468), and cellular components such as nucleus (GO:0005634), cytosol (GO:0005829), and methionine/threonine/tyrosine kinase activity (GO:0005634) and cytosol (GO:0005829). (GO:0005634), cellular components such as cytosol (GO:0005829), and MFs such as methionine serine/threonine/tyrosine kinase activity (GO:0004712) and enzyme binding (GO:0019899).

**Figure 6.**
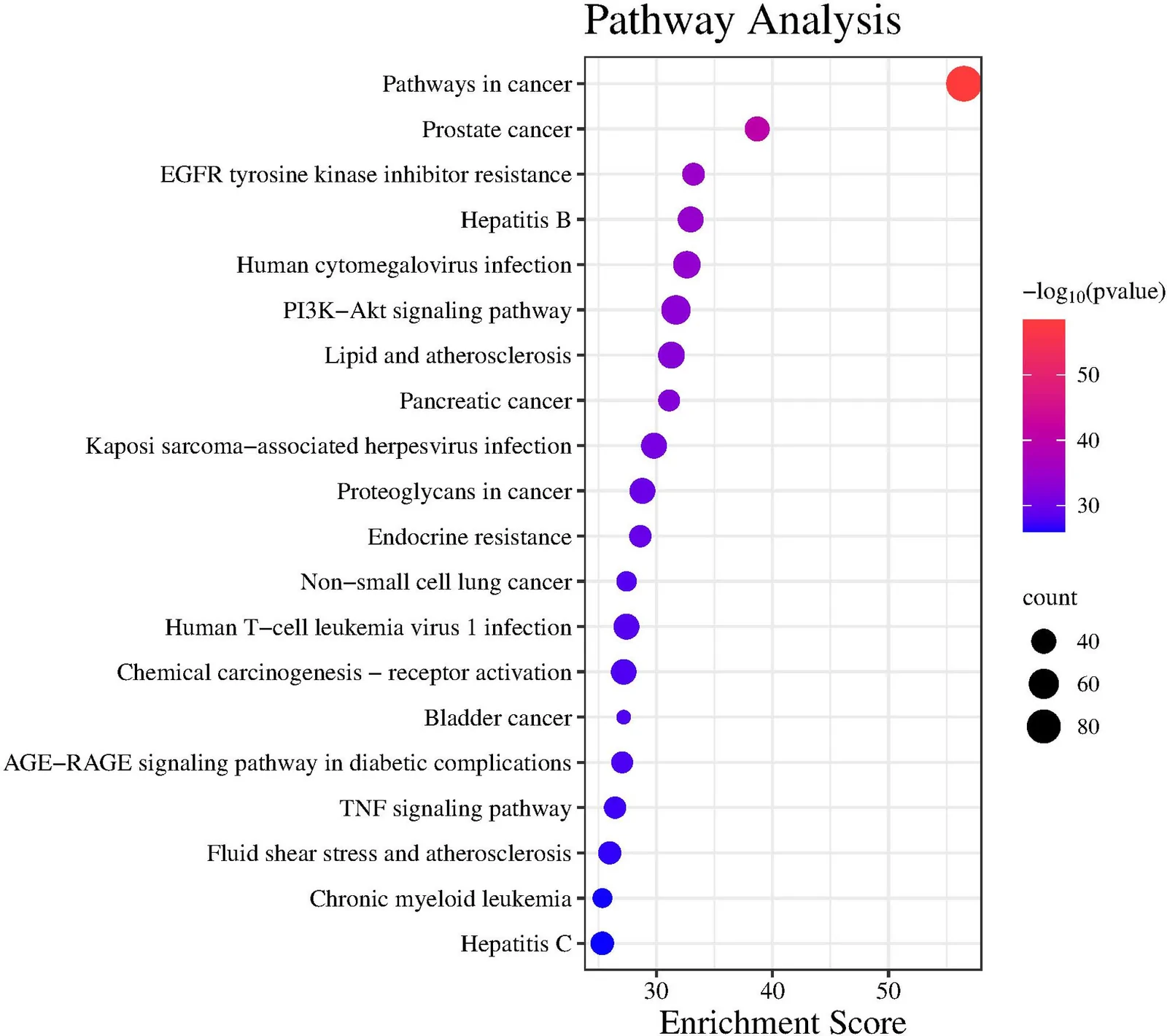
Significantly enriched bubble chart through visualization.

KEGG pathway was applied to explore the function and signaling pathways of KLX anti-NSCLC targets. There were 178 statistically significant KLX-NSCLC-related pathways, of which the top 20 pathways, selected based on a significance threshold of *P* < .05, were visualized by the number of key targets are shown in Figure 6. The results showed that KLX is involved in the pathway through the development of cancer (hsa05200), prostate cancer (hsa05215), and resistance to epidermal growth factor receptor tyrosine kinase inhibitors (hsa01521), hepatitis B (hsa05161), human cytomegalovirus infection (hsa05163), and non-small cell lung cancer pathway (hsa05223).

**Figure 7.**
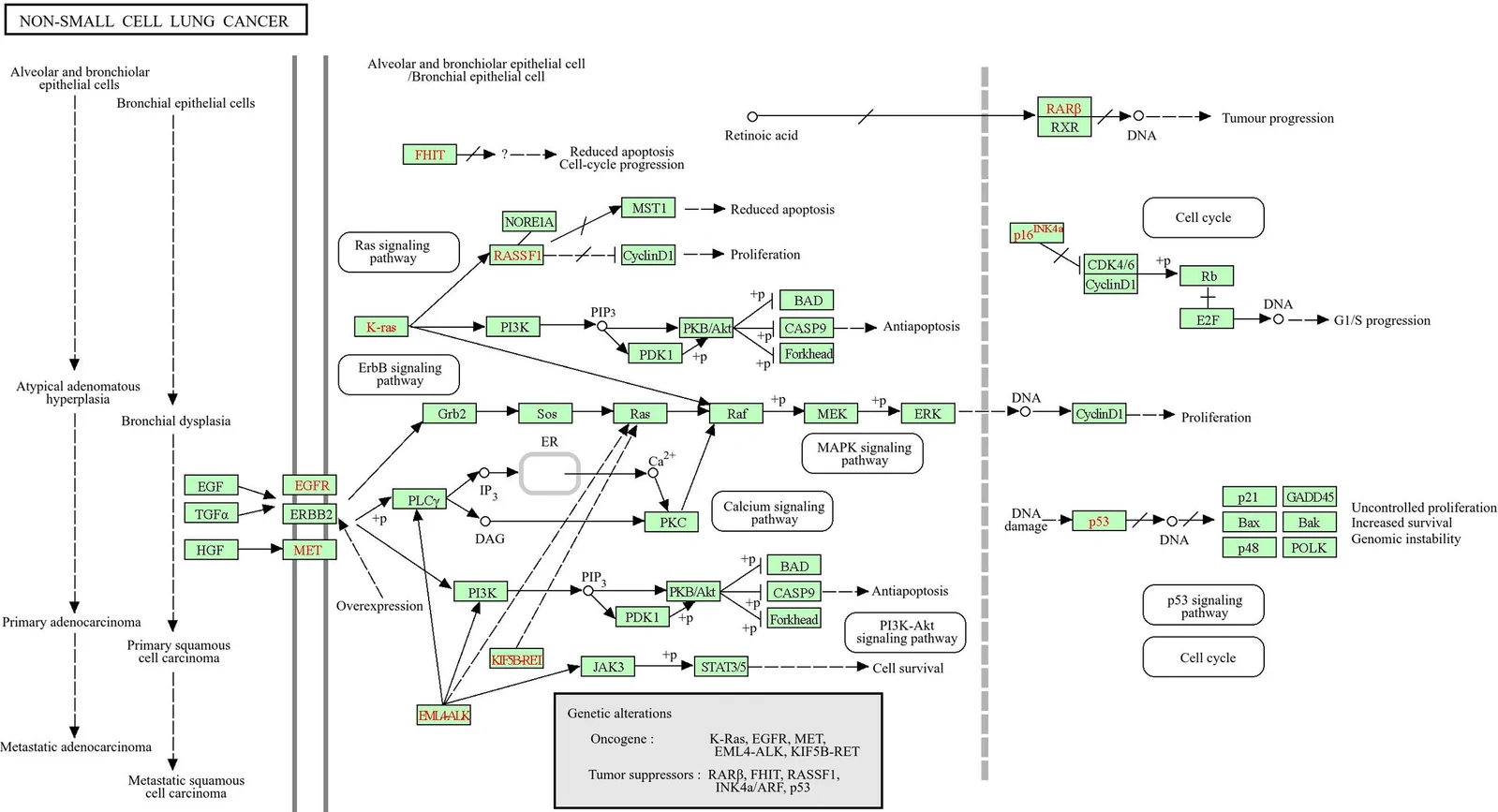
KEGG mapping of non-small cell lung cancer pathways. KEGG = Kyoto encyclopedia of genes and genomes.

In conclusion, KLX can play an important role in the treatment of NSCLC through multiple targets and pathways. In this study, the non-small cell lung cancer pathway (hsa05223, Fig. 7) was considered to be the most important pathway of KLX in the treatment of NSCLC.

### 3.6. Docking of molecules

**Figure 8.**
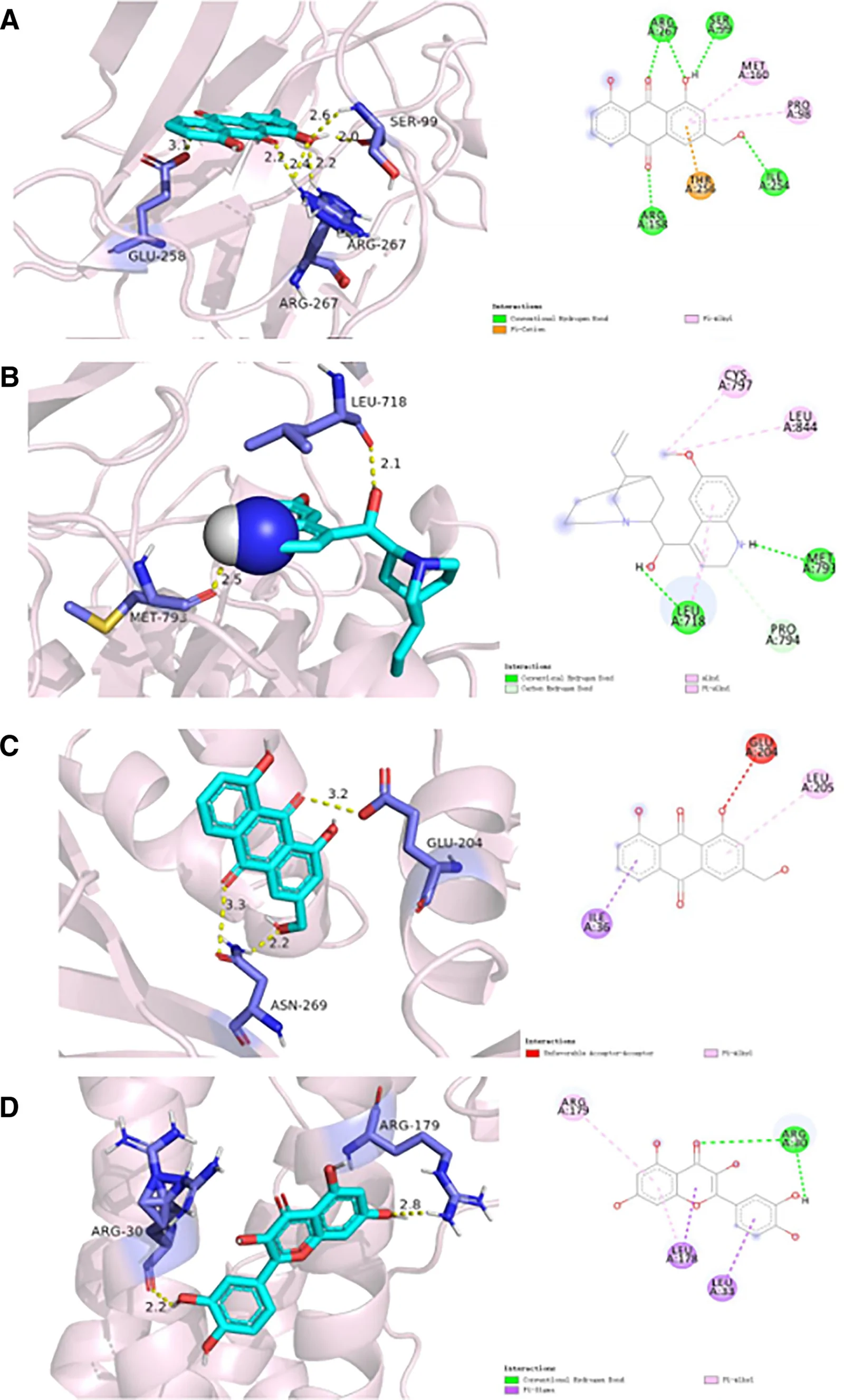
Four docking models for affinity < −7.0 kcal/mol. (A) TP53 versus aloe-emodin, (B) EGFR versus HZ02, (C) MYC versus aloe-emodin, and (D) IL6 versus quercetin.

After docking the top 10 core target proteins with their KLX counterparts (Table 3), the binding energies <−7.0 kcal/mol were found to be the binding energies of TP53 and aloe-emodin, EGFR and HZ02 ((R)-(6-methoxy-4-quinolyl)-[(2R,4R,5S)-5 vinylquinuclidin-2-yl]methanol), MYC and aloe-emodin, IL6 and quercetin, and IL6 and quercetin–(R-(6-methoxy-4-quinolyl)-[(2R,4R,5S)-5-vinylquinuclidin-2-yl]methanol), MYC with aloe-emodin, and IL6 with quercetin. The results of molecular docking are shown in Figure 8. EGFR binds most stably to HZ02 (binding energy −8.6 kcal/mol), mainly through the formation of salt-bridged hydrogen bonding interactions with the target proteins of HZ02, such as MET-793 and LEU-718, and the formation of hydrophobic interactions of Pi-alkyl with CYS-797 and LEU-844. Together, these interactions enhance the stability of the complex. At the same time, these components can bind well to the active site of the core target and show strong binding activity. To validate these findings, we compared our docking energy values with known inhibitors. For example, EGFR inhibitors typically exhibit binding energies in the range of −7 to −13 kcal/mol, suggesting that our identified compounds may have strong binding affinity relative to established inhibitors. TP53 inhibitors report binding affinities between −7 and −11 kcal/mol indicating that our active ingredients exhibit binding within a comparable range. MYC inhibitors typically exhibit binding energies in the range of –8 to −15 kcal/mol. IL-6 inhibitors typically exhibit binding energies in the range of −8 to −12 kcal/mol. The results showed that the 3 representative compounds in KLX, aloe rhodopsin, quercetin and HZ02, could exert therapeutic effects by binding to the 4 core target proteins, including TP53 of NSCLC.

**Table 3:** The binding energy between the top 10 core targets and their corresponding components.

Target protein	Corresponding active compound	Binding energy (kcal/mol)
TP53	Aloe-emodin	−8.3
GAPDH	Adenosine	−6.8
EGFR	(R)-(6-Methoxy-4-quinolyl)-[(2R,4R,5S)-5-vinylquinuclidin-2-yl] methanol	−8.6
AKT1	Kaempferol	−6.0
	Quercetin	−6.3
CASP3	Arachidonic acid	−4.8
	Beta-sitosterol	−7.0
MYC	Aloe-emodin	−7.4
BCL2	Beta-sitosterol	−6.4
TNF	Aspongopusamideb	−6.7
	Aloe-emodin	−6.5
	Kaempferol	−6.8
	Quercetin	−7.0
IL6	Quercetin	−7.1
PTEN	Quercetin	−6.5

In conclusion, Aloe barbadensis, quercetin, and HZ02, the representative compounds in KLX, as well as the signaling pathways and BPs involved in KLX-NSCLC, play important roles in the pathogenesis and treatment of NSCLC. Network pharmacology and molecular docking studies provide a theoretical basis and potential mechanism for KLX treatment of NSCLC.

## 4. Discussion

### 4.1. Quercetin, aloe rhodopsin and the non-small cell lung cancer pathway

Studies have shown that an important derivative of aloe-emodin, aloe-emodin-3-(hydroxymethyl)-O-β-D-glucopyranoside (AE3G), significantly inhibits the growth of NSCLC cells while exhibiting low toxicity to normal cells such as Vero cells. AE3G, while exhibiting low toxicity to normal cells (e.g., Vero cells), significantly inhibited the growth of NSCLC cells.^[5]^ This property is mainly attributed to the inhibitory effect of AE3G on MEK/ERK and Akt signaling pathways, as well as its ability to induce mitochondria-mediated apoptosis, thus effectively interfering with the growth mechanism of NSCLC cells.

Aloe-emodin, a natural compound extracted from aloe vera, has been noted for its significant antitumor, antimicrobial, and immunomodulatory activities.^[6]^ Although its specific mechanism of action in NSCLC has not been fully clarified, based on its wide range of anticancer activities, it can be hypothesized that aloe-emodin may affect NSCLC by inhibiting the proliferation of cancer cells and inducing apoptosis through 2 main pathways. On the one hand, it may inhibit the growth of tumor cells by interfering with the basic biosynthesis of DNA, RNA, and proteins; on the other hand, it may use the programmed cell death mechanism to accurately eliminate cancer cells in the body.^[7]^

Therefore, aloe-emodin is not only excellent in inhibiting the proliferation of NSCLC cells, but also enhances its anticancer effect by inducing apoptosis, which provides a new way of thinking and a potential solution for the treatment of NSCLC. This finding not only highlights the great potential of aloe-emodin in the field of medicine, but also points out the direction for the development of anticancer drugs in the future.

On the other hand, quercetin, as a polyphenolic compound widely found in nature, has shown a unique role in cancer therapy, especially in affecting the growth and apoptosis of NSCLC cell lines, by virtue of its abundant biological activities, such as antioxidant, anti-inflammatory, antifibrotic, and antiviral properties.^[8]^ It has been shown that its core mechanism is to promote autophagy and finely regulate SIRT1 and AMPK signaling pathways, which have profound effects on the fate of NSCLC cells.^[9]^

In NSCLC treatment research, EGFR as a key target has attracted much attention. With its unique targeting ability, quercetin can directly inhibit the activity of EGFR and then block the downstream signaling, significantly inhibiting the proliferation, and migration of NSCLC cells. This discovery provides a new approach for the treatment of NSCLC. Further, quercetin has shown the potential to promote the degradation of EGFR mutants (e.g., drug resistance-associated T790M mutation), which can help to restore the sensitivity of patients to tyrosine kinase inhibitors and provide a new strategy to address the problem of drug resistance in NSCLC.^[10]^

In addition, quercetin has shown good synergism with chemotherapeutic agents, especially in combination with drugs such as gefitinib, which can significantly enhance the therapeutic effect on NSCLC. This synergistic mechanism may involve the enhancement of the antitumor activity of chemotherapeutic drugs or the alleviation of their side effects, providing strong support for the comprehensive treatment of NSCLC.^[11]^ However, pharmacokinetic studies have shown that aloe emodin has poor intestinal absorption, a short elimination half-life, and low bioavailability.^[12]^

In conclusion, aloe-emodin and quercetin, with their unique mechanisms of multi-pathway and multi-targeting, have shown a broad potential in the treatment of NSCLC. They not only provide novel ideas and diversified choices for the clinical treatment of NSCLC, but also profoundly reveal the great value of these 2 natural compounds in anticancer research. These important discoveries not only further strengthen the foundation of the research and development of aloe-emodin and quercetin as anticancer drugs, but also open up a new path for the exploration of new anticancer drugs as well as the innovation of NSCLC treatment strategies in the future, pointing out the way forward.

### 4.2. Association of TP53, EGFR, MYC, and IL6 with the non-small cell lung cancer pathway

The importance of several core genes and signaling pathways in the treatment of NSCLC cannot be ignored. Among them, TP53 (p53), as a key tumor suppressor gene, is essential for maintaining cell cycle regulation, inducing apoptosis and DNA repair.^[13]^ However, mutations in TP53 are very common in NSCLC, especially in smokers. These mutations lead to the loss of p53 function, which prevents the malignant transformation of damaged cells and promotes tumor development. Therefore, it is important to understand the mutation status of TP53 for treatment selection and prognosis of NSCLC patients.

EGFR also plays an important role in the evolution of NSCLC. When EGFR is abnormally activated or overexpressed, it significantly enhances the proliferation, survival and migration of tumor cells, reduces the dependence on growth factors, and enables tumor cells to maintain vigorous growth in a hostile environment. This abnormal activation also exacerbates the invasiveness and metastasis of tumor cells and accelerates the deterioration of the disease.^[14]^ Therefore, EGFR-targeted therapies play an important role in the treatment of NSCLC. By inhibiting the activity of EGFR or blocking its binding to ligands, the growth and invasion of tumor cells can be effectively curbed, which can bring better therapeutic effects to patients.

In addition to TP53 and EGFR, the MYC gene also plays an important role in the pathogenesis of NSCLC, and its overexpression promotes tumor cell proliferation and survival, and interacts with other key signaling pathways to participate in the immune evasion mechanism, enabling tumor cells to successfully evade the surveillance and attack of the immune system. Such aberrant expression may also affect the sensitivity of NSCLC cells to drugs, thus reducing the therapeutic effect.^[15]^ Therefore, targeted therapeutic strategies against MYC are receiving widespread attention and are expected to become a new treatment option for NSCLC patients.

In addition, IL-6 also plays an important role in the process of NSCLC, and the expression level of IL-6 is closely related to the malignancy of NSCLC, which affects tumor cell proliferation, apoptosis, angiogenesis and invasive migration by binding to the receptor and activating downstream signaling pathways.^[16]^ In order to inhibit IL-6-mediated tumor growth and metastasis, researchers are actively exploring antibody therapies targeting IL-6 or its receptor, which are expected to provide new strategies for the treatment of NSCLC.

However, it is important to recognize that NSCLC progression is a complex and variable process that involves the interaction of multiple factors and signaling pathways. Therefore, the treatment of NSCLC needs to be individualized, taking into account the patient’s specific situation and the severity of the disease. With the help of network pharmacology and molecular docking, we can better understand the role of these key genes and signaling pathways in NSCLC, and provide more precise and effective strategies for clinical treatment.

### 4.3. Current treatment research for NSCLC and limitations of KLX

In recent years, targeted therapies have been developed for the epidermal growth factor receptor, anaplastic lymphoma kinase, Rat C-ROS oncogene 1 receptor tyrosine kinase RET, human epidermal growth factor receptor-2, BRAF, MET, KRAS, and other targets, clinical research has achieved remarkable results. Breakthroughs in the molecular mechanisms of next-generation targeted drugs have enabled more precise treatment, significantly improving therapeutic outcomes. Meanwhile, combination therapy strategies for targeted drugs have also demonstrated significant efficacy. By combining different targeted drugs with chemotherapy, radiotherapy, or other targeted drugs, these strategies not only enhance synergistic effects but also effectively overcome drug resistance issues, providing NSCLC patients with additional treatment options.^[17]^ Brigatinib is a reversible dual inhibitor of ALK and the epidermal growth factor receptor, and is effective against ALK, wild-type EGFR, L858R/T790M-resistant EGFR mutations, c-ros oncogene 1 receptor tyrosine kinase, FMS-like tyrosine kinase 3, and insulin-like growth factor 1 receptor.^[18]^ This drug can block ALK-mediated downstream signaling pathways such as JAK/STAT, MAPK, or PI3K/AKT, ultimately inhibiting tumor cell proliferation and survival.^[19]^ Anemarrhena asphodeloides’ active ingredients decreased AKT1, SRC, and HSP90AA1 expression and suppressed the malignant phenotype of human NSCLC cells.^[20]^ KLX acts on the targets TP53, EGFR, MYC, and IL-6 and has good development potential.

Despite these promising findings, several limitations must be acknowledged. The study does not address the bioavailability of KLX active compounds, which is a critical factor in drug development. Herbal compounds often suffer from poor absorption, rapid metabolism, or low systemic circulation, which can limit their therapeutic potential. Future research should conduct pharmacokinetic studies to determine the stability, absorption, and metabolic fate of these compounds in vivo. Our findings are based on in silico and in vitro analyses, and further validation through in vivo animal models and clinical trials is necessary to establish the clinical relevance of KLX. Animal studies should assess its pharmacodynamics, dose–response relationships, and efficacy in tumor regression. While our study suggests that KLX exhibits anticancer effects, a comprehensive assessment of its potential side effects is lacking. Many herbal compounds have been associated with off-target effects, hepatotoxicity, or immunomodulatory activity. Future investigations should evaluate its toxicity profile, potential drug interactions, and long-term safety.

## Acknowledgments

We are appreciated for all the public databases and websites applied in the present study.

## Author contributions

**Conceptualization:** Mingxuan Li.

**Data curation:** Yilong Li.

**Formal analysis:** Yilong Li.

**Methodology:** Xu Gong.

**Software:** Kangwei Xia, Zhaorui Liu.

**Supervision:** Xiangjun Qiu.

**Writing – original draft:** Yilong Li.

**Writing – review & editing:** Xiangjun Qiu.

## References

[1] Nooreldeen R Bach H. Current and future development in lung cancer diagnosis. Int J Mol Sci . 2021;22:8661. 10.3390/ijms22168661 PMC8395394 34445366

[2] Huang Y Xia L Tan X. Molecular mechanism of lncRNA SNHG12 in immune escape of non-small cell lung cancer through the HuR/PD-L1/USP8 axis. Cell Mol Biol Lett. 2022;27:43. 10.1186/s11658-022-00343-7 PMC9164758 35658874

[3] Wang J Quan Y. Clinical study on Kanglixin capsules combined with chemotherapy in treatment of advanced non-small cell lung cancer. Chin J Mod Med. 2018;28:73–6.

[4] Liu J Liu J Tong X. Network pharmacology prediction and molecular docking-based strategy to discover the potential pharmacological mechanism of huai hua san against ulcerative colitis. Drug Des Devel Ther. 2021;15:3255–76. 10.2147/DDDT.S319786 PMC8326529 34349502

[5] Kim HJ Choi JW Ree J. Aloe emodin 3-O-glucoside inhibits cell growth and migration and induces apoptosis of non-small-cell lung cancer cells via suppressing MEK/ERK and Akt signalling pathways. Life Sci. 2022;300:120495. 10.1016/j.lfs.2022.120495 35341826

[6] Luo H Ji X Zhang M. Aloe-emodin: progress in pharmacological activity, safety, and pharmaceutical formulation applications. Mini Rev Med Chem. 2024;24:1784–98. 10.2174/0113895575298364240409064833 38639277

[7] Chen S Liu C Xiao S Tang J. Research progress on anti-tumor mechanism of aloe-emodin. Mod Pharm Clin Remedies. 2022;37:1150–55.

[8] Deng X Chen D Xie A. Quercetin alleviates hyperoxia-induced bronchopulmonary dysplasia by inhibiting ferroptosis through the MAPK/PTGS2 pathway: Insights from network pharmacology, molecular docking, and experimental evaluations. Chem Biol Drug Des. 2024;103:e14520. 10.1111/cbdd.14520 38570710

[9] Guo H Ding H Tang X. Quercetin induces pro-apoptotic autophagy via SIRT1/AMPK signaling pathway in human lung cancer cell lines A549 and H1299 in vitro. Thorac Cancer. 2021;12:1415–22. 10.1111/1759-7714.13925 PMC8088950 33709560

[10] Ge Z Xu M Ge Y. Inhibiting G6PD by quercetin promotes degradation of EGFR T790M mutation. Cell Rep. 2023;42:113417. 10.1016/j.celrep.2023.113417 37950872

[11] Tiwari H Karki N Pal M. Functionalized graphene oxide as a nanocarrier for dual drug delivery applications: the synergistic effect of quercetin and gefitinib against ovarian cancer cells. Colloids Surf B Biointerfaces. 2019;178:452–9. 10.1016/j.colsurfb.2019.03.037 30921680

[12] Dong X Zeng Y Liu Y. Aloe-emodin: a review of its pharmacology, toxicity, and pharmacokinetics. Phytother Res. 2020;34:270–81. 10.1002/ptr.6532 31680350

[13] Wang M Li P Wan R Liu X. Integrated analysis of the prognostic value of TP53 dependent etoposide-induced gene 24 in non-small cell lung cancer. Biomed Pharmacother. 2019;112:108590. 10.1016/j.biopha.2019.01.051 30784913

[14] Nagasaka M Zhu VW Lim SM Greco M Wu F Ou SI. Beyond osimertinib: the development of third-generation EGFR tyrosine kinase inhibitors for advanced EGFR+ NSCLC. J Thorac Oncol. 2021;16:740–63. 10.1016/j.jtho.2020.11.028 33338652

[15] Lei T Xu T Zhang N. Anlotinib combined with osimertinib reverses acquired osimertinib resistance in NSCLC by targeting the c-MET/MYC/AXL axis. Pharmacol Res. 2023;188:106668. 10.1016/j.phrs.2023.106668 36681369

[16] Liu W Wang H Bai F. IL-6 promotes metastasis of non-small-cell lung cancer by up-regulating TIM-4 via NF-κB. Cell Prolif. 2020;53:e12776. 10.1111/cpr.12776 PMC7106962 32020709

[17] Liu Y Zhao J. Research progress of targeted therapy for non-small cell lung cancer in 2024. J Comprehensive Cancer Ther. 2025;11:52–60.

[18] Hoy SM. Brigatinib: a review in ALK-inhibitor naïve advanced ALK-positive NSCLC. Drugs. 2021;81:267–75. 10.1007/s40265-020-01449-y 33528789

[19] Kiełbowski K Żychowska J Becht R. Anaplastic lymphoma kinase inhibitors – a review of anticancer properties, clinical efficacy, and resistance mechanisms. Front Pharmacol. 2023;14:1285374. 10.3389/fphar.2023.1285374 PMC10634320 37954850

[20] Xie X Jiang Y Liu S Xie C. Integrating molecular docking and network pharmacology to reveal the molecular mechanisms of *Anemarrhena asphodeloides* in the treatment of non-small cell lung cancer. Discov Oncol. 2025;16:1399. 10.1007/s12672-025-03178-8 PMC12287501 40702358

